# Supplementary material for: Holocene reconfiguration and readvance of the East Antarctic Ice Sheet
Source: Nat Commun. 2018 Aug 9;9:3176. doi: 10.1038/s41467-018-05625-3 (PMC6085394; doi:10.1038/s41467-018-05625-3)
Supplement: Supplementary file 1 — Supplementary Information [file 41467_2018_5625_MOESM1_ESM.pdf]

**Supplementary Information:**

**Holocene reconfiguration and readvance of the East Antarctic Ice Sheet, Greenwood *et al*.**

---

Sarah L. Greenwood[1*], Lauren M. Simkins[2,3], Anna Ruth W. Halberstadt[2,4], Lindsay O. Prothro[2], John B. Anderson[2]

*[1]Department of Geological Sciences, Stockholm University, Stockholm 10691, Sweden*

*[2]Department of Earth, Environmental and Planetary Sciences, Rice University, Houston, TX 77005, USA*

*[3]Department of Environmental Sciences, University of Virginia, Charlottesville, VA 22904, USA*

*[4]Department of Geosciences, University of Massachusetts, Amherst, MA 01003, USA*

**Corresponding author: sarah.greenwood@geo.su.se*

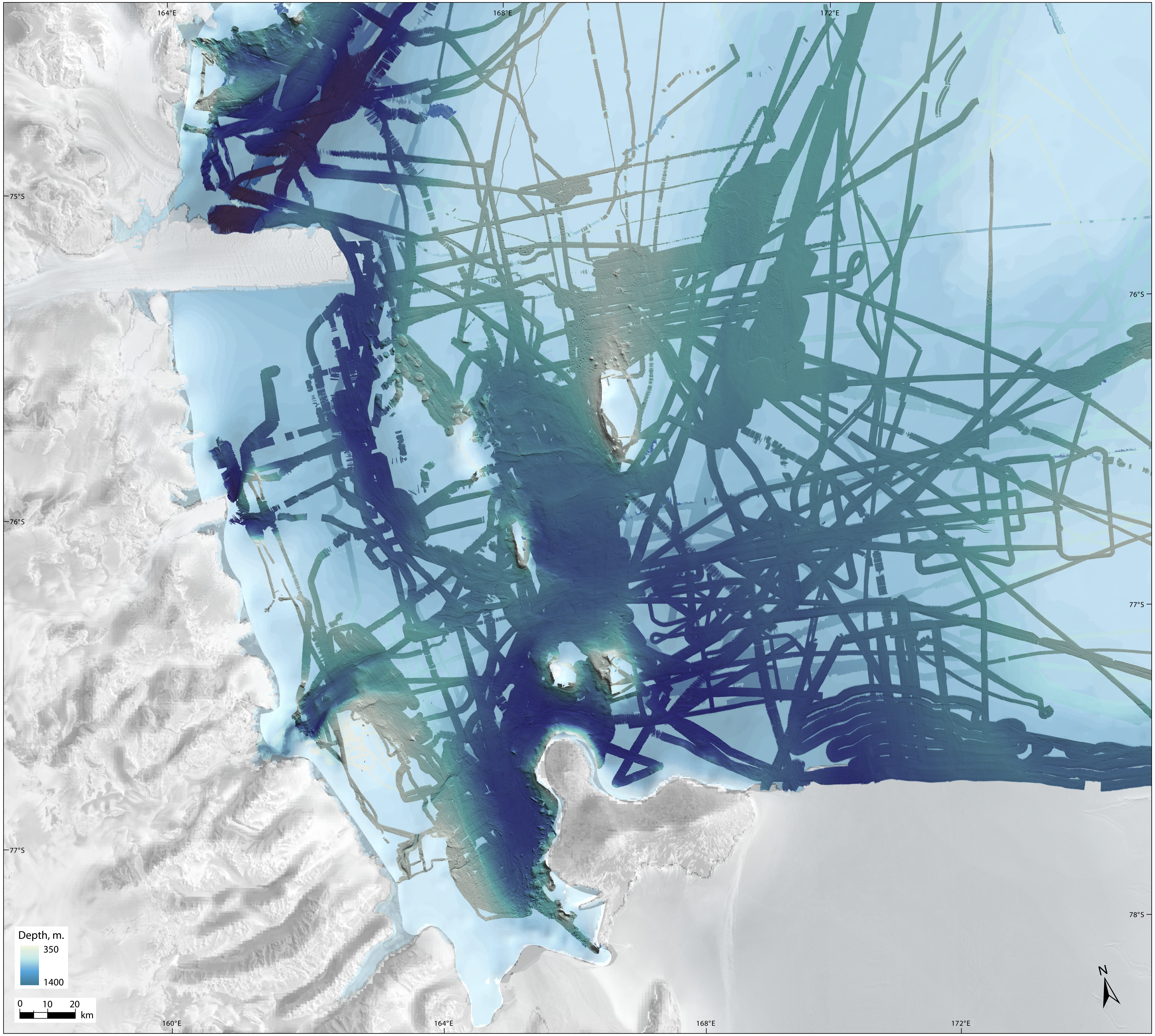


Supplementary Fig. 1. Compilation of multibeam data in the western Ross Sea. Kongsberg EM120 & 122 data in full colour, visualised with 4x vertical exaggeration and hillshade from the NE; legacy SeaBeam data semi-transparent. Background data from IBCSO[1] and RAMP[2] ice surface mosaic.

**Supplementary Table 1**: Estimates bracketing likely duration of Grounding Zone Wedge construction.

| | | GZW1 | | Whole GZW complex | |
|---|---|---|---|---|---|
| *Acoustic velocity through sediment* | | *1500 m s$^{-1}$* | *1750 m s$^{-1}$* | *1500 m s$^{-1}$* | *1750 m s$^{-1}$* |
| Grounding line length (km) | | 34.5 | | -- | |
| GZW area (km$^2$) | | 679.42 | | 1062.08 | |
| Mean GZW thickness (m) | | 4.39 | 5.12 | 3.35 | 3.91 |
| GZW volume (km$^3$) | | 2.98 | 3.48 | 4.06 | 4.74 |
| **Duration of GZW stillstand (years)** | | | | | |
| - flux: | 100 m$^3$ a$^{-1}$ m$^{-1}$ | 865 | 1009 | 1396 | 1628 |
| | 500 m$^3$ a$^{-1}$ m$^{-1}$ | 173 | 202 | 280 | 326 |
| | 1000 m$^3$ a$^{-1}$ m$^{-1}$ | 86 | 101 | 140 | 163 |

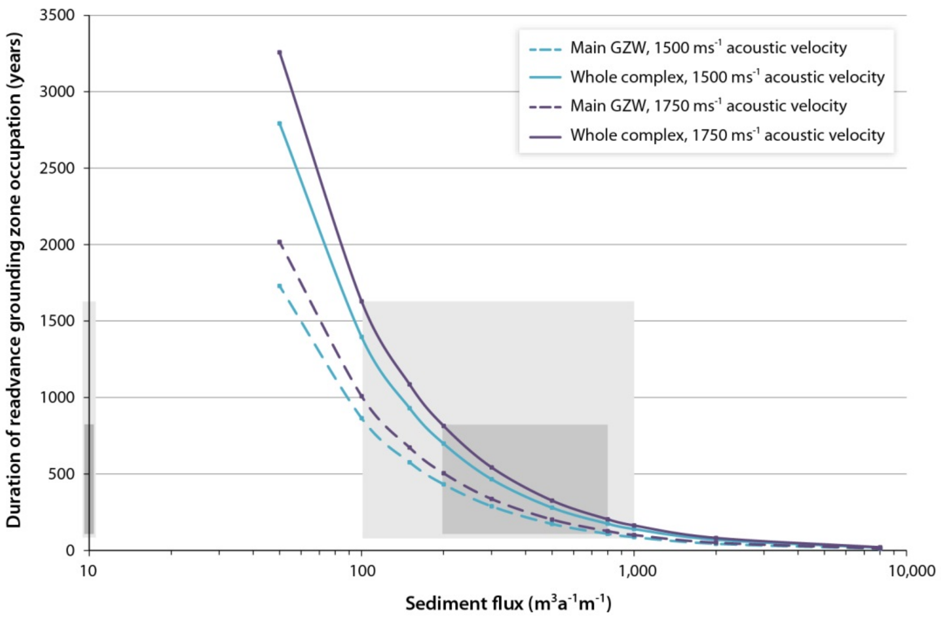


**Supplementary Fig. 2**: Duration of Grounding Zone Wedge construction for varying grounding line sediment fluxes. Light grey envelope denotes fluxes of 100-1000 m$^3$a$^{-1}$m$^{-1}$, consistent with most literature reported values[3-9], yielding event durations of c. 80-1000 years; a slightly narrower range of fluxes (200-800 m$^3$a$^{-1}$m$^{-1}$; dark grey envelope) yields durations of c. 100-800 years.

**Supplementary Table 2**: Estimated ice volume discharged by the southern JOIDES readvance event (Fig. 4G, 7A), using an event duration of 100-1600 years (Supplementary Table 1, Supplementary Fig. 2) and potential ice flow velocities bracketed by 200 and 800 m $a^{-1}$.

| Duration (years) | Velocity (m $a^{-1}$) | Flowline length passing across readvance maximum grounding line (km) | Method: parabola with no isostatic correction | Ice volume ($km^3$) | Annual discharge (Gt) |
|---|---|---|---|---|---|
| *100* | *200* | *20 -> too short* | -- | -- | |
| 100 | 500 | 50 | c = 2.0 | 3560 | 32.6 |
| 100 | 800 | 80 | c = 2.0 | 7591 | 69.6 |
| 600 | 200 | 120 | c = 2.0 | 10119 | 15.5 |
| 600 | 500 | 300 | c = 2.0 | 26461 | 40.4 |
| 600 | 800 | 480 | c = 2.0 | 42196 | 64.5 |
| 1600 | 200 | 320 | c = 2.0 | 26894 | 15.4 |
| | | | | | |
| 100 | 500 | 50 | c = 2.5 | 3831 | 35.1 |
| 100 | 800 | 80 | c = 2.5 | 8254 | 75.7 |
| 600 | 200 | 120 | c = 2.5 | 11073 | 16.9 |
| 600 | 500 | 300 | c = 2.5 | 30814 | 47.1 |
| 600 | 800 | 480 | c = 2.5 | 54002 | 82.5 |
| 1600 | 200 | 320 | c = 2.5 | 31678 | 18.2 |
| *1600* | *500* | *800 -> catchment uncertain* | *volume scaled up* | *~87000* | *49.9* |
| *1600* | *800* | *1280 -> catchment uncertain* | *volume scaled up* | *~152000* | *87.1* |
| | | | | | |
| 100 | 500 | 50 | c = 3.0 | 4103 | 37.6 |
| 100 | 800 | 80 | c = 3.0 | 8918 | 81.8 |
| 600 | 200 | 120 | c = 3.0 | 12026 | 18.4 |
| 600 | 500 | 300 | c = 3.0 | 35300 | 53.9 |
| 600 | 800 | 480 | c = 3.0 | 66413 | 101.5 |
| 1600 | 200 | 320 | c = 3.0 | 36701 | 21.0 |
| | | | | | |
| | | **Grounding line advance (km)** | **Method: parabola with no isostatic correction** | **Total discharge (Gt)** | |
| | | 50 | c = 2.0 | 3265 | |
| | | 50 | c = 2.5 | 3513 | |
| | | 50 | c = 3.0 | 3762 | |

**Supplementary Table 3:** Multibeam data compiled from archives (www.marine-geo.org and oden.geo.su.se). *Newly acquired.

| Cruise | System | PI | doi | Cruise | System | PI | doi |
|---|---|---|---|---|---|---|---|
| NBP9407 | SeaBeam2112 | Bartek | 10.1594/IEDA/100366 | NBP0409 | EM120 | Anderson | 10.1594/IEDA/100323 |
| NBP9501 | SeaBeam2112 | Anderson | 10.1594/IEDA/100328 | NBP0501 | EM120 | Gordon | 10.1594/IEDA/100391 |
| NBP9601 | SeaBeam2112 | Luyendyk, Bartek | 10.1594/IEDA/100376 | NBP0602 | EM120 | Stock, Cande | 10.1594/IEDA/100287 |
| NBP9602 | SeaBeam2112 | Cande | 10.1594/IEDA/100285 | NBP0701 | EM120 | Cande | 10.1594/IEDA/100374 |
| NBP9702 | SeaBeam2112 | Cande | 10.1594/IEDA/100337 | NBP0702 | EM120 | Nitsche | 10.1594/IEDA/100372 |
| NBP9801 | SeaBeam2112 | Anderson | 10.1594/IEDA/100346 | NBP0801 | EM120 | Caron | 10.1594/IEDA/100383 |
| NBP9902 | SeaBeam2112 | Anderson | 10.1594/IEDA/100355 | NBP1005 | EM120 | Yager | 10.1594/IEDA/100400 |
| NBP0001 | SeaBeam2112 | Jacobs | 10.1594/IEDA/100296 | NBP1101 | EM120 | Kohut | 10.1594/IEDA/317589 |
| NBP0209 | EM120 | Cande | 10.1594/IEDA/100314 | NBP1201 | EM120 | McGillycuddy | 10.1594/IEDA/319324 |
| NBP0301 | EM120 | Luyendyk | 10.1594/IEDA/100315 | NBP1210 | EM120 | Halanych | 10.1594/IEDA/320750 |
| NBP0301A | EM120 | Bart | 10.1594/IEDA/100316 | NBP1302 | EM120 | Hansell | 10.1594/IEDA/320074 |
| NBP0301B | EM120 | Smith | 10.1594/IEDA/100317 | NBP1502A* | EM122 | Anderson | 10.1594/IEDA/321969 |
| NBP0302 | EM120 | Gordon | 10.1594/IEDA/100318 | OSO0708 | EM120 | Swedish Polar Research Secretariat | -- |
| NBP0305A | EM120 | Smith | 10.1594/IEDA/100288 | OSO0910 | EM122 | Anderson, Jakobsson | -- |
| NBP0306 | EM120 | Luyendyk, Bartek | 10.1594/IEDA/100289 | OSO1011 | EM122 | Swedish Polar Research Secretariat | -- |
| NBP0401 | EM120 | Wilson | 10.1594/IEDA/100381 | Araon2013 | EM122 | Lee | 10.1594/IEDA/323884 |
| NBP0402 | EM120 | Visbeck | 10.1594/IEDA/100368 | Araon2015 | EM122 | Lee | 10.1594/IEDA/323884 |

**Supplementary References**


1. Arndt, J.E., *et al.* The International Bathymetric Chart of the Southern Ocean (IBCSO) Version 1.0—A new bathymetric compilation covering circum-Antarctic waters. *Geophysical Research Letters* **40**, 3111-3117 (2013).
2. Jezek, K.C., *et al.* RAMP AMM-1 SAR image mosaic of Antarctica. National Snow and Ice Data Center, Boulder, Colorado, USA. https://doi.org/10.5067/8AF4ZRPULS4H.
3. Hooke, R.L. & Elverhøi, A. Sediment flux from a fjord during glacial periods, Isfjorden, Spitsbergen. *Global and Planetary Change* **12,** 237-249 (1996).
4. Kamb, B. Basal zone of the West Antarctic ice streams and its role in lubrication of their rapid motion. *Antarctic Research Series* **77,** 157-199 (2001).
5. Bougamont, M. & Tulaczyk, S. Glacial erosion beneath ice streams and ice-stream tributaries: constraints on temporal and spatial distribution of erosion from numerical simulations of a West Antarctic ice stream. *Boreas* **32,** 178-190 (2003).
6. Ó Cofaigh, C., Evans, J., Dowdeswell, J.A. & Larter, R.D. Till characteristics, genesis and transport beneath Antarctic paleo-ice streams. *Journal of Geophysical Research, Earth Surface* **112,** F03006 (2007).
7. Anandakrishnan, S., Catania, G., Alley, R.B. & Horgan, H. Discovery of till deposition at the grounding line of Whillans ice stream. *Science* **315,** 1835-1838 (2007).
8. Christoffersen, P., Tulaczyk, S. & Behar, A. Basal ice sequences in Antarctic ice stream: Exposure of past hydrologic conditions and a principal mode of sediment transfer. *Journal of Geophysical Research, Earth Surface* **115,** F03034 (2010).
9. Golledge, N. R. *et al.* Glaciology and geological signature of the Last Glacial Maximum Antarctic ice sheet. *Quaternary Science Reviews* **78,** 225–247 (2013).
